# Supplementary material for: Screening of Drosophila microRNA-degradation sequences reveals Argonaute1 mRNA’s role in regulating miR-999
Source: Nat Commun. 2023 Apr 13;14:2108. doi: 10.1038/s41467-023-37819-9 (PMC10102002; doi:10.1038/s41467-023-37819-9)
Supplement: Supplementary file 1 — Supplementary Information [file 41467_2023_37819_MOESM1_ESM.pdf]

Supplementary Figure 1

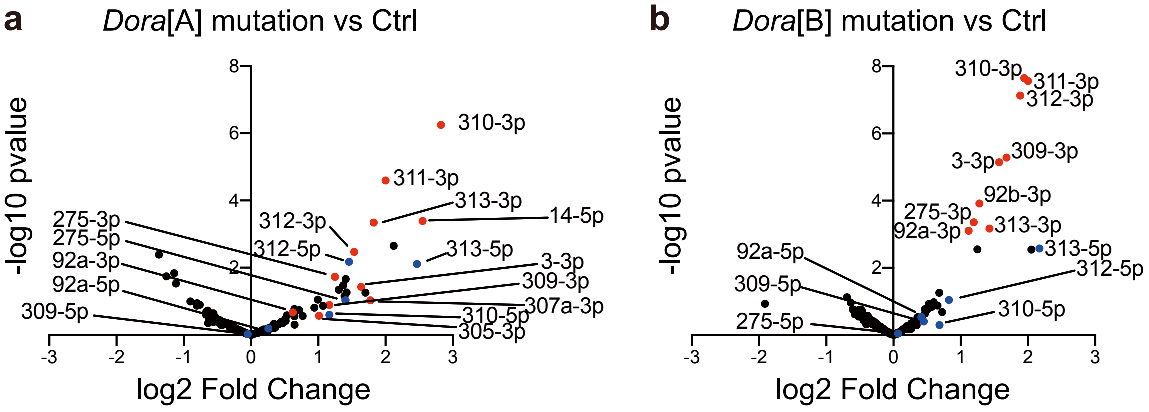

Supplementary Fig. 1 miRNA abundance in embryos with *Dora* mutations. Changes in miRNA abundance observed from *Dora*[A] (**a**) or *Dora*[B] (**b**) embryos compared with control embryos by small RNA-seq. Guide strands of the *Dora*-sensitive miRNAs are indicated by red dots, and the blue dots represent their passenger strands. Source data are provided as a Source Data file.

Supplementary Figure 2

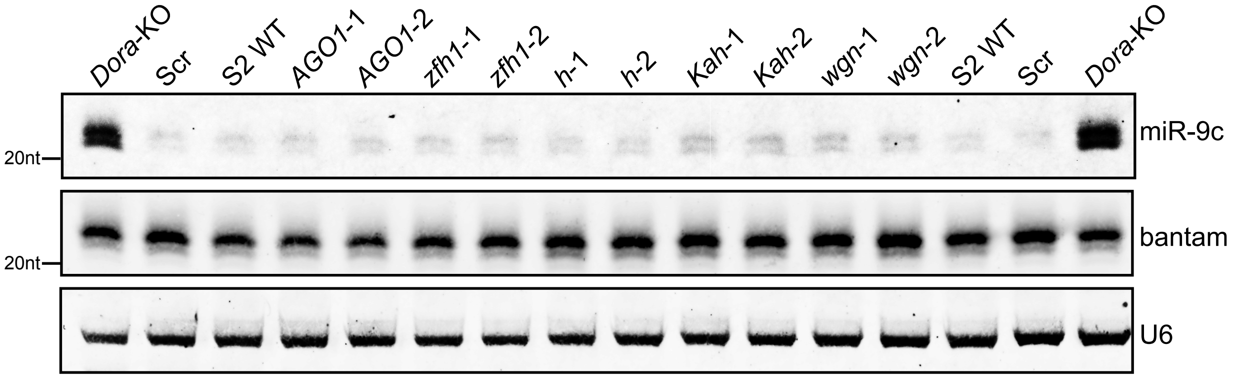

Supplementary Fig. 2 Northern blot analyses of miR-9c in TDMD trigger knockout cells. Northern blots detecting miR-9c, bantam and U6 in TDMD trigger knockout of *AGO1*, *zfh1*, *h*, *Kah*, *wgn* and WT, control-KO (Scr), *Dora* KO S2 cells. Total RNAs were prepared as described in Fig. 3a. Source data are provided as a Source Data file.

Supplementary Figure 3

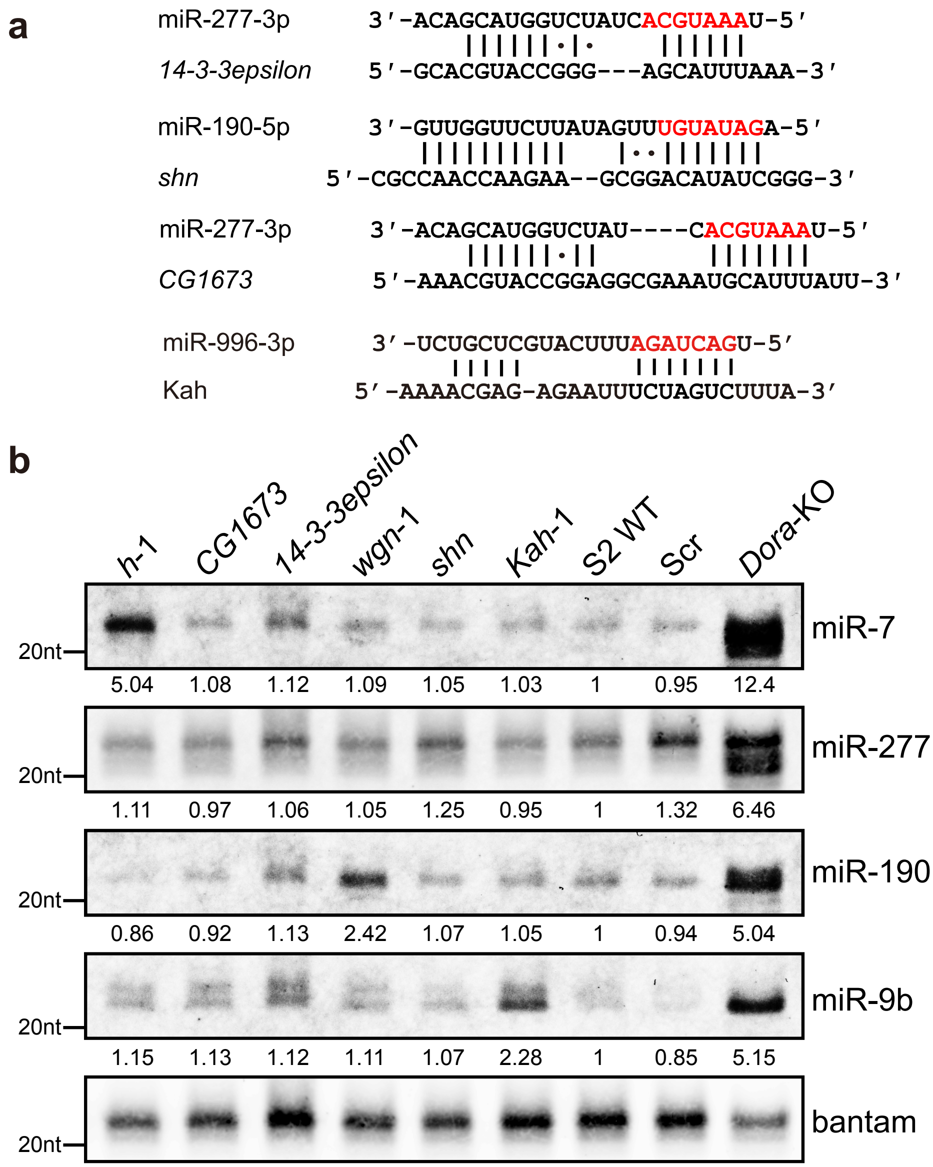

Supplementary Fig. 3 Knockout of low-confidence TDMD trigger in S2 cells. a Base-pairing pattern of miRNAs and potential TDMD triggers. Red letters represent miRNA seed region. **b** Northern blot analyses of miR-7, miR-277, miR-190, miR-9b, bantam and U6 in TDMD trigger knockout of *h*, *CG1673*, *14-3-3epsilon*, *wgn*, *shn*, *Kah* and WT, control-KO (Scramble), *Dora*-KO S2 cells. Total RNAs were extracted from each TDMD trigger knockout population cells selected with 5 µg/mL puromycin for 4 weeks. The levels of bantam serve as a loading control. *h*/miR-7, *Kah*/miR-9b and *wgn*/miR-190 serve as positive TDMD pairs. The miRNA abundance normalized to bantam was shown below each miRNA. The miRNA abundance in WT was normalized to 1. n=3 biological replicates. Source data are provided as a Source Data file.

Supplementary Figure 4

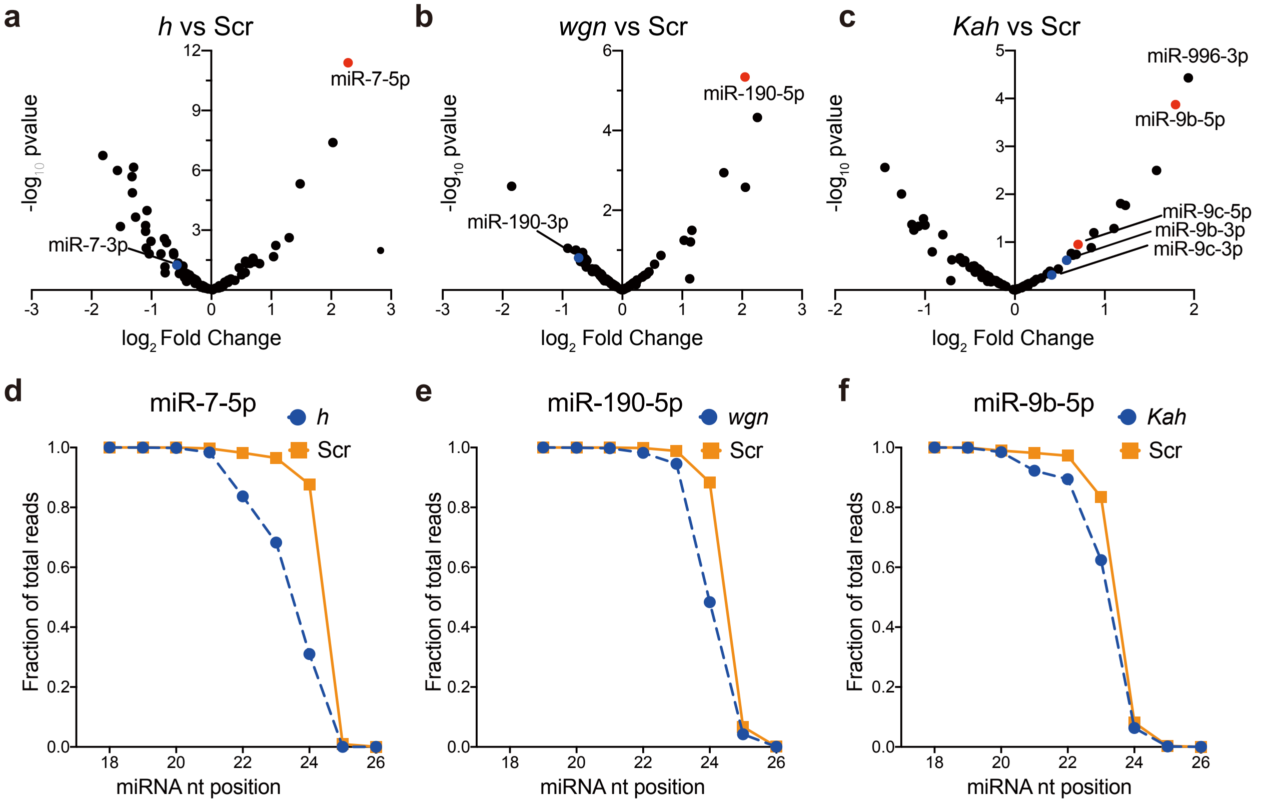

Supplementary Fig. 4 TDMD triggers influence miRNA abundance, 3′ end extension. The miRNA abundance change detected by small RNA-seq in *h* (**a**), *wgn* (**b**) and *Kah* (**c**) TDMD trigger KO cells compared with control-KO (Scramble) cells. miR-7, miR-190 and miR-9b are indicated by red dots, and the blue dots represent their passenger strands. The fraction of small RNA-seq reads with coverage of 18-26 nucleotides (nt) for miR-7 (**d**), miR-190 (**e**) and miR-9b (**f**). For each miRNA, solid lines delineate the control KO samples, dash lines delineate the TDMD trigger KO samples. Data are presented as mean ± SD. n=2 biological replicates. Source data are provided as a Source Data file.

Supplementary Figure 5

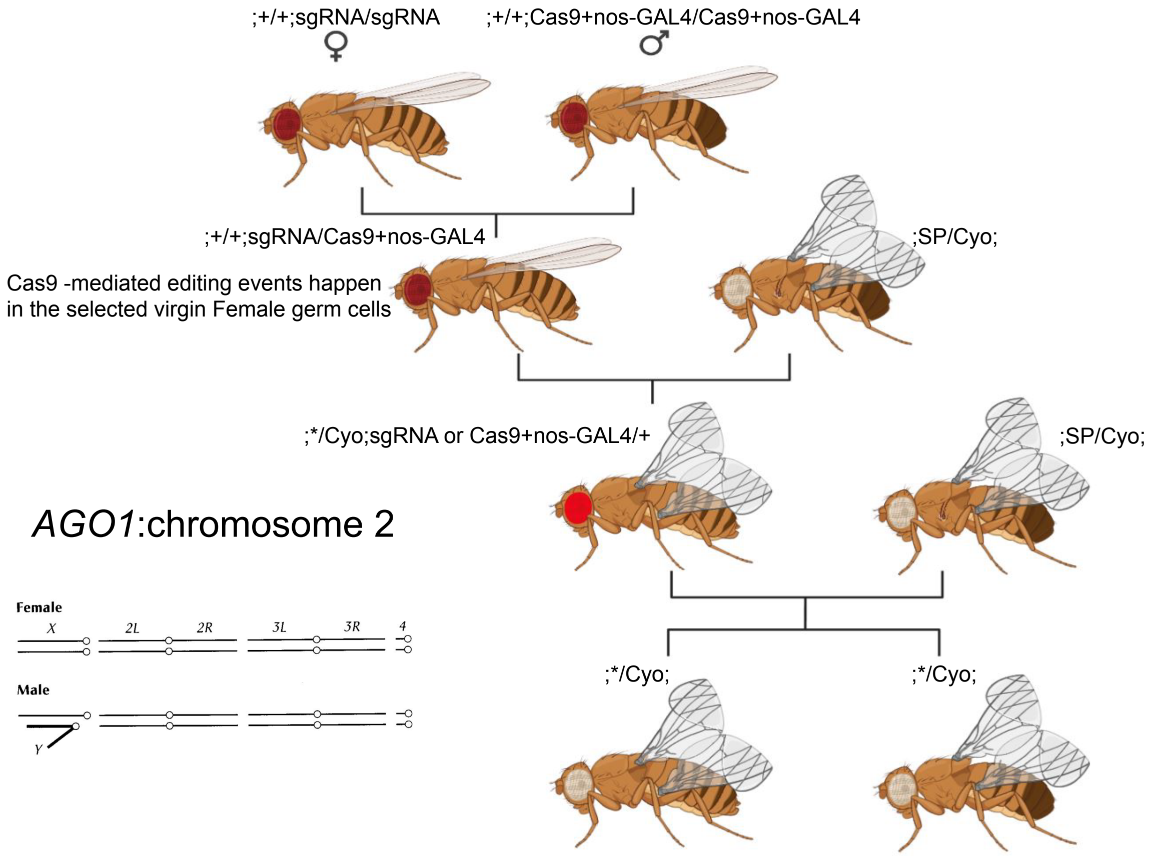

Supplementary Fig. 5 Schematic showing the generation of *AGO1* trigger KO flies. Virgin female flies containing *AGO1* trigger-targeting CRISPR-Cas9 in the germ cells were crossed twice with Cyo balancer flies to obtain potential lines containing the *AGO1* trigger deletion.* represents mutation of the *AGO1* trigger. Cartoons were created with BioRender.com.

Supplementary Figure 6

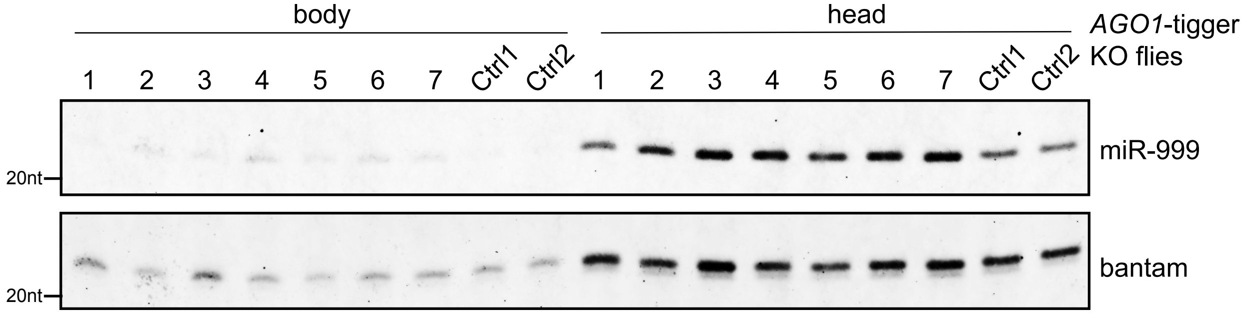

Supplementary Fig. 6 Northern blot analyses of miR-999 in *AGO1* trigger deletion flies. Total RNAs were extracted from the head and body of 30 flies. Samples of head and body were loaded at 20 and 30 ug total RNA, respectively. Bantam serves as a loading control. n=3 biological replicates. Source data are provided as a Source Data file.

Supplementary Figure 7

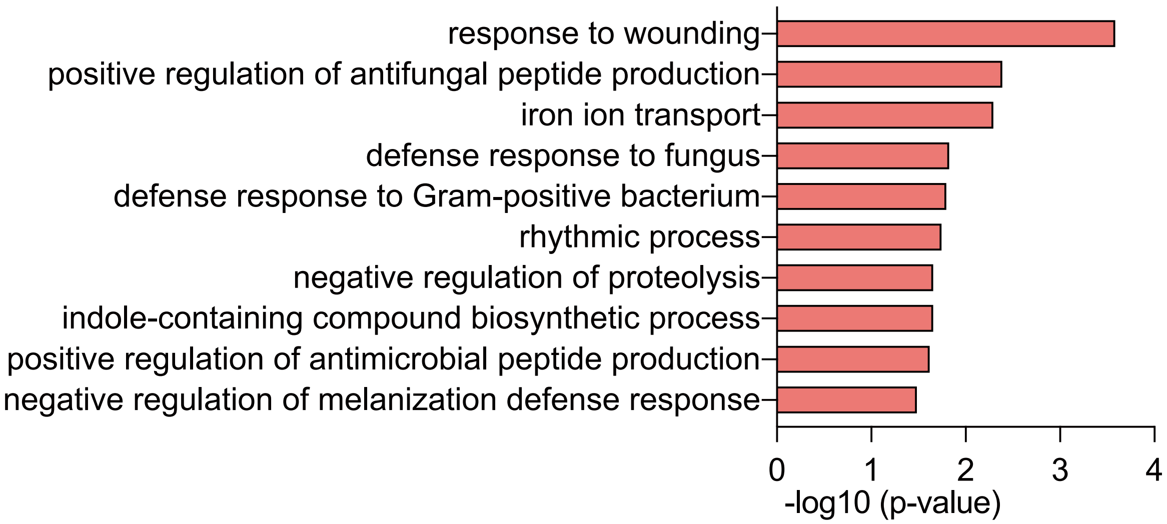

Supplementary Fig. 7 Biological functions of down-regulated genes in *AGO1* trigger KO S2 cells. DAVID identified GO term biological pathways enriched in down-regulated genes in *AGO1* trigger-KO compared with control-KO S2 cells. Unadjusted p-values were determined for the GO term analysis. Source data are provided as a Source Data file.

Supplementary Figure 8

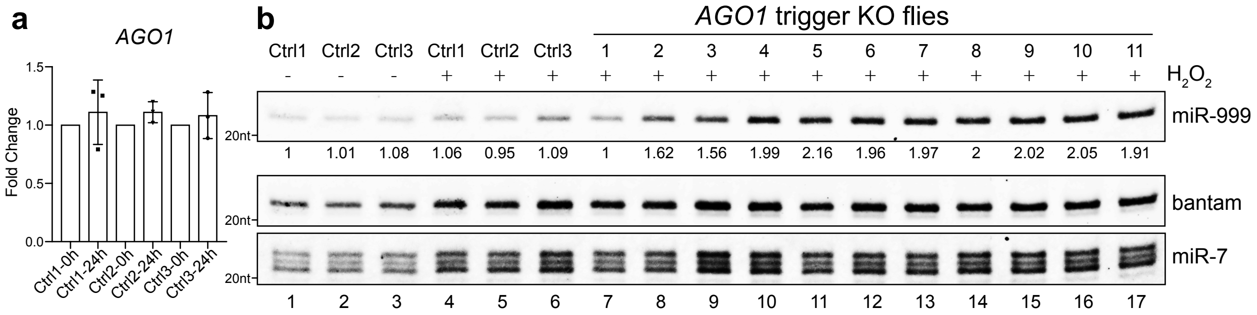

Supplementary Fig. 8 Levels of *AGO1* and miR-999 in flies treated with H_2O_2. a RT-qPCR analyses of *AGO1* mRNA level in H_2O_2 treated and untreated control flies, normalized to *Actin*. The *AGO1* abundance in each untreated control lines were normalized as 1. Data are presented as mean ± SD. n=3 biological replicates. **b** Northern blot analyses of miR-999, bantam and miR-7 in *AGO1* trigger deletion and control flies treated with H_2O_2. Bantam and miR-7 served as loading controls. The normalized miR-999 abundance (compared to bantam) are shown below each miRNA band. The miRNA abundance in untreated control line no.1 was normalized as 1. n=3 biological replicates. Source data are provided as a Source Data file.

Supplementary Figure 9

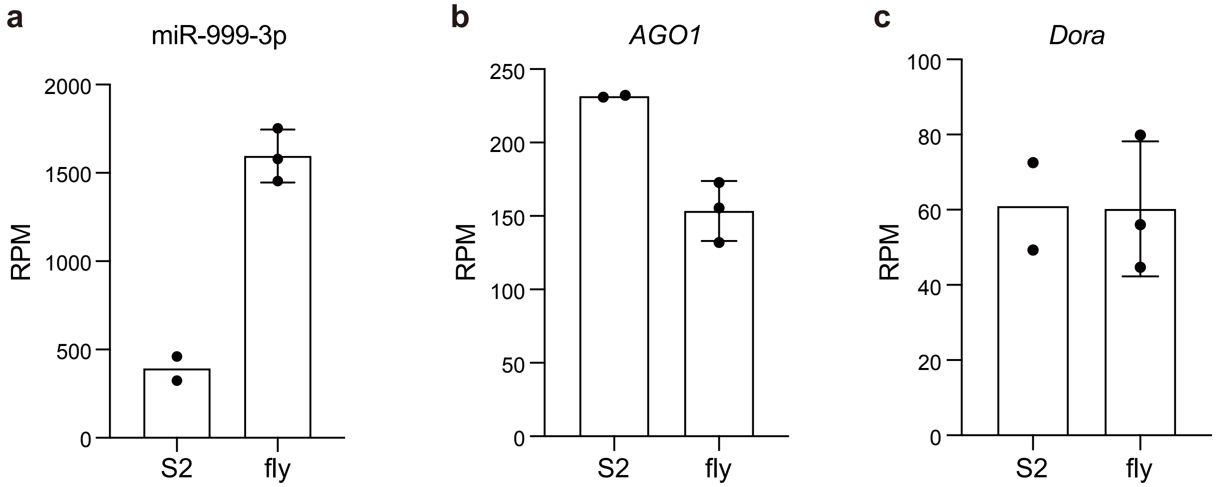

Supplementary Fig. 9 miR-999, *AGO1*, and *Dora* levels in control S2 cells and flies. The abundance of miR-999 (**a**), *AGO1* (**b**) and *Dora* (**c**) are measured in RNA-sequencing experiments performed in control knockout S2 cells and flies. Data are presented from 2 or 3 biological replicates. Error bars represent SD. The expression values are represented in RPM (reads per million). Source data are provided as a Source Data file.

Supplementary Figure 10

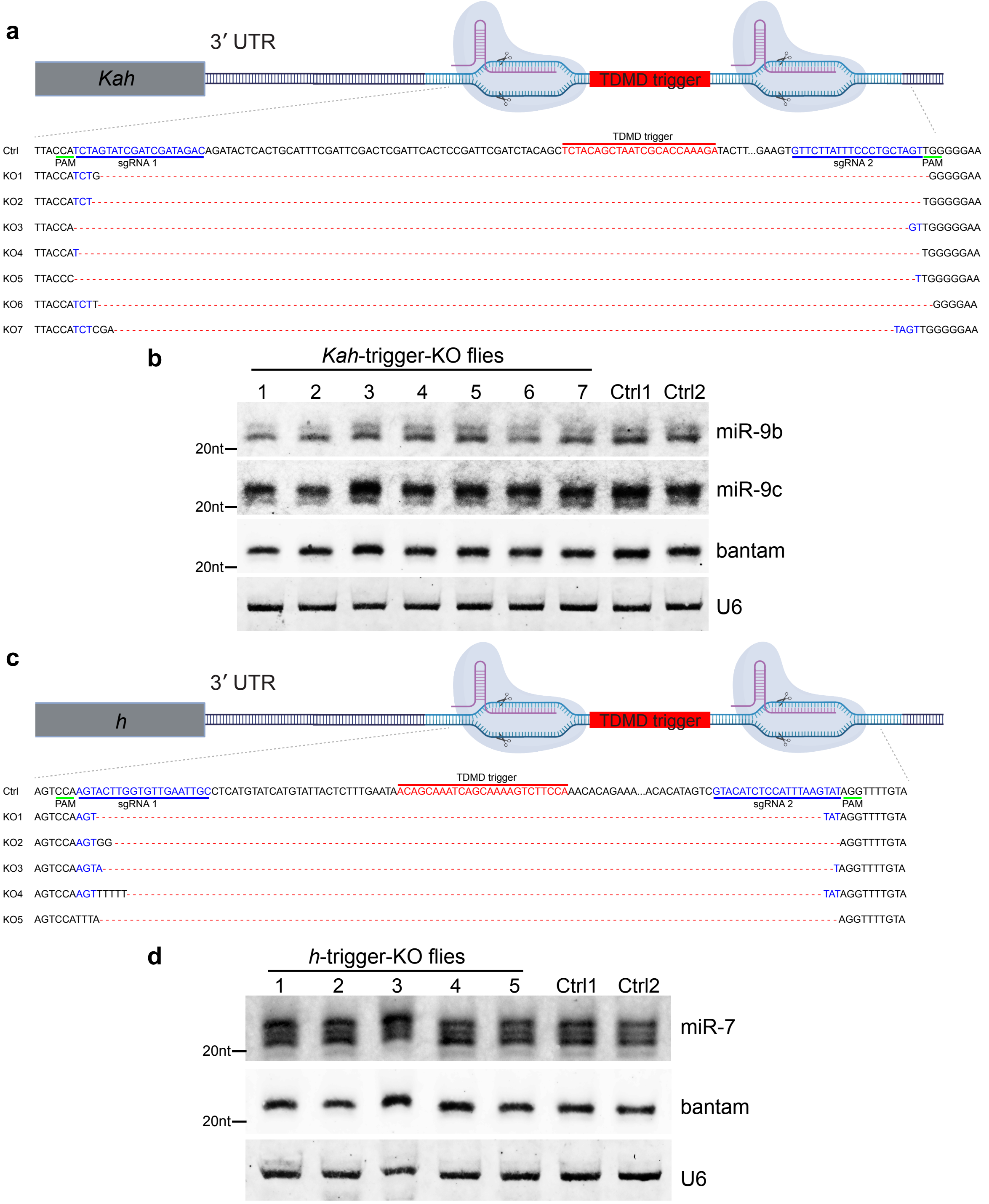

Supplementary Fig. 10 Deletion of *Kah* and *h* TDMD trigger in *Drosophila*. Schematic of the CRISPR-Cas9-mediated mutation of TDMD trigger from *Kah* 3′ UTR (**a**) and *h* 3′UTR (**c**). Cartoons were created with BioRender.com. The TDMD trigger and sgRNA sites are highlighted in red and blue, respectively. PAM sequences are underlined (green). The genotype of the control line and each mutant line is shown below. Northern blot analyses detect miR-9b (**b**), miR-7 (**d**), bantam and U6 in control lines and TDMD trigger mutant lines. Bantam and U6 serve as loading controls. n=3 biological replicates. Source data are provided as a Source Data file.
