# Supplementary material for: Screening of Drosophila microRNA-degradation sequences reveals Argonaute1 mRNA’s role in regulating miR-999
Source: Nat Commun. 2023 Apr 13;14:2108. doi: 10.1038/s41467-023-37819-9 (PMC10102002; doi:10.1038/s41467-023-37819-9)
Supplement: Supplementary file 3 — Description of Additional Supplementary Files [file 41467_2023_37819_MOESM3_ESM.pdf]

Description of Additional Supplementary Files

File Name: Supplementary Data 1
Description: List of candidate TDMD hybrids in Drosophila S2 cells.

File Name: Supplementary Data 2
Description: List of adapters for sequencing library processing and oligonucleotides for plasmid construction, PCR, northern blot and morpholino.

File Name: Supplementary Data 3
Description: DAVID identified GO term biological pathways enriched in down-regulated genes in AGO1 trigger KO S2/flies compared with control.

File Name: Supplementary Data 4
Description: Survival rate for AGO1 trigger KO flies after hydrogen peroxide exposure compared with control-KO flies.
