# Supplementary material for: Screening of Drosophila microRNA-degradation sequences reveals Argonaute1 mRNA’s role in regulating miR-999
Source: Nat Commun. 2023 Apr 13;14:2108. doi: 10.1038/s41467-023-37819-9 (PMC10102002; doi:10.1038/s41467-023-37819-9)
Supplement: Supplementary file 8 — Reporting Summary [file 41467_2023_37819_MOESM8_ESM.pdf]

nature portfolio

Corresponding author(s): Lu Li, Lei Zhou, Mingyi Xie

Last updated by author(s): 03-18-2023

Reporting Summary

Nature Portfolio wishes to improve the reproducibility of the work that we publish. This form provides structure for consistency and transparency in reporting. For further information on Nature Portfolio policies, see our Editorial Policies and the Editorial Policy Checklist.

Statistics

For all statistical analyses, confirm that the following items are present in the figure legend, table legend, main text, or Methods section.

n/a	Confirmed	
☐	☒	The exact sample size (*n*) for each experimental group/condition, given as a discrete number and unit of measurement
☐	☒	A statement on whether measurements were taken from distinct samples or whether the same sample was measured repeatedly
☐	☒	The statistical test(s) used AND whether they are one- or two-sided<br>*Only common tests should be described solely by name; describe more complex techniques in the Methods section.*
☒	☐	A description of all covariates tested
☒	☐	A description of any assumptions or corrections, such as tests of normality and adjustment for multiple comparisons
☐	☒	A full description of the statistical parameters including central tendency (e.g. means) or other basic estimates (e.g. regression coefficient) AND variation (e.g. standard deviation) or associated estimates of uncertainty (e.g. confidence intervals)
☐	☒	For null hypothesis testing, the test statistic (e.g. *F*, *t*, *r*) with confidence intervals, effect sizes, degrees of freedom and *P* value noted<br>*Give P values as exact values whenever suitable.*
☒	☐	For Bayesian analysis, information on the choice of priors and Markov chain Monte Carlo settings
☒	☐	For hierarchical and complex designs, identification of the appropriate level for tests and full reporting of outcomes
☐	☒	Estimates of effect sizes (e.g. Cohen's *d*, Pearson's *r*), indicating how they were calculated

Our web collection on statistics for biologists contains articles on many of the points above.

Software and code

Policy information about availability of computer code

Data collection	Northern blot images were obtained by Amersham Typhoon Scanner with ImageQuant TL (v7.0) software. RT-qPCR data were obtained by Bio-Rad CFX96 real-time PCR machine with the Bio-Rad CFX Maestro 2.0 software.
Data analysis	ImageQuant is used for analyzing irNorthern blot signals obtained from Amersham Typhoon scanner. GraphPad Prism8 is used for statistical analysis. For bioinformatic analysis, all custom scripts have been made available at https://github.com/UF-Xie-Lab/TDMD-in-Drosophila. Go term were analyzed by DAVID Bioinformatics Resource v2022q3 (https://david.ncifcrf.gov).The adapters from the poly-A RNA-seq reads were removed using Cutadapt (version 3.4), and the clean reads were mapped to the Drosophila melanogaster genome (FlyBase Release 6.32) using Hisat2 (version 2.2.1). Gene counts were calculated using the HTSeq-count software (version 0.11.1). The DESeq2 package (version 1.20.0) in R was utilized to identify differentially expressed genes. Cumulative fraction curves (CFCs) were generated using the matplotlib library (version 3.4.1) and a custom Python script, 'CLASH.py'.

For manuscripts utilizing custom algorithms or software that are central to the research but not yet described in published literature, software must be made available to editors and reviewers. We strongly encourage code deposition in a community repository (e.g. GitHub). See the Nature Portfolio guidelines for submitting code & software for further information.

Data

Policy information about availability of data

All manuscripts must include a data availability statement. This statement should provide the following information, where applicable:

- Accession codes, unique identifiers, or web links for publicly available datasets
- A description of any restrictions on data availability
- For clinical datasets or third party data, please ensure that the statement adheres to our policy

All custom scripts can be accessed through our GitHub repository at https://github.com/UF-Xie-Lab/TDMD-in-Drosophila and the Zenodo repository at https://doi.org/10.5281/zenodo.7737958. Additional modified scripts can be accessed upon request. All sequencing data that support the findings of this study have been deposited in the National Center for Biotechnology Information Sequence Read Archive (SRA) and are accessible through the BioProject PRJNA896239.

Human research participants

Policy information about studies involving human research participants and Sex and Gender in Research.

Reporting on sex and gender	n/a
Population characteristics	n/a
Recruitment	n/a
Ethics oversight	n/a

Note that full information on the approval of the study protocol must also be provided in the manuscript.

Field-specific reporting

Please select the one below that is the best fit for your research. If you are not sure, read the appropriate sections before making your selection.

☒ Life sciences ☐ Behavioural & social sciences ☐ Ecological, evolutionary & environmental sciences

For a reference copy of the document with all sections, see nature.com/documents/nr-reporting-summary-flat.pdf

Life sciences study design

All studies must disclose on these points even when the disclosure is negative.

Sample size	AGO1-CLASH libraries were separated and size selected between 147 and 527 bp as previously described.Fields CJ, et al. Sequencing of Argonaute-bound microRNA/mRNA hybrids reveals regulation of the unfolded protein response by microRNA-320a. PLoS genetics 17, e1009934 (2021).Since the size of free adaptor is 127bp and the size of miRNA is about 20, the minimum library needs 147bp. The 527bp size was chosen because the size of the library was mostly below 527bp.
Data exclusions	No data were excluded
Replication	Three AGO1-CLASH libraries were generated from control-KO and Dora-KO S2 cells respectively.<br>All mRNA and sRNA sequencing experiments were performed in duplicate.<br>All attempts at replication were successful for all experiments.
Randomization	Different knockout and control Drosophila lines were grouped based on genotype.
Blinding	The investigators were not blind to the flies' genotype, but data collection and analysis was done in an automated fashion.

Reporting for specific materials, systems and methods

We require information from authors about some types of materials, experimental systems and methods used in many studies. Here, indicate whether each material, system or method listed is relevant to your study. If you are not sure if a list item applies to your research, read the appropriate section before selecting a response.

Materials & experimental systems

n/a	Involved in the study
☐	☒ Antibodies
☐	☒ Eukaryotic cell lines
☒	☐ Palaeontology and archaeology
☐	☒ Animals and other organisms
☒	☐ Clinical data
☒	☐ Dual use research of concern

Methods

n/a	Involved in the study
☒	☐ ChIP-seq
☒	☐ Flow cytometry
☒	☐ MRI-based neuroimaging

Antibodies

Antibodies used	Anti-AGO1 antibody (Abcam #ab5070),AGO1-IP was carried out with magnetic protein A dynabeads (Life Technologies) (100 µl) conjugated with 20 ug Anti-AGO1 antibody,diluted with 1:200.
Validation	Anti-AGO1 antibody validated in Wessels H-H, et al. Global identification of functional microRNA-mRNA interactions in Drosophila. Nature communications 10, 1-12 (2019).

Eukaryotic cell lines

Policy information about cell lines and Sex and Gender in Research

Cell line source(s)	Dr. David P. Bartel for kindly providing WT, Scramble and Dora-KO S2 cells.<br>All the TDMD triggers KO cell lines were established in the WT S2 cells using CRISPR-Cas9 system.
Authentication	none
Mycoplasma contamination	The cell lines were not tested for mycoplasma contamination.
Commonly misidentified lines (See ICLAC register)	none

Animals and other research organisms

Policy information about studies involving animals; ARRIVE guidelines recommended for reporting animal research, and Sex and Gender in Research

Laboratory animals	Flies expressing sgRNAs were generated by Rainbow Transgenic Flies,detailed in the manuscript Methods.<br>Stocks obtained from Bloomington Drosophila Stock Center:<br>Two lines with Dora mutation stock number 52333 and 52334<br>Flies expressing Cas9 stock number 54593<br>All the flies used were about 1 week old adults.
Wild animals	No wild animals were used in this study.
Reporting on sex	All northern blot in flies were random used male and female mix flies. Oxidative Stress Assays only used male for the experiment.
Field-collected samples	No Field-collected samples were used in this study.
Ethics oversight	No ethics oversight is required for Drosophila studies.

Note that full information on the approval of the study protocol must also be provided in the manuscript.
